# Supplementary material for: Chemical Evaluation of Liquidambar styraciflua L. Fruits Extracts and Their Potential as Anticancer Drugs
Source: Molecules. 2023 Jan 1;28(1):360. doi: 10.3390/molecules28010360 (PMC9822488; doi:10.3390/molecules28010360)
Supplement: Supplementary file 1 [file molecules-28-00360-s001.zip › molecules-2104438-supplementary.pdf]

Supplementary material

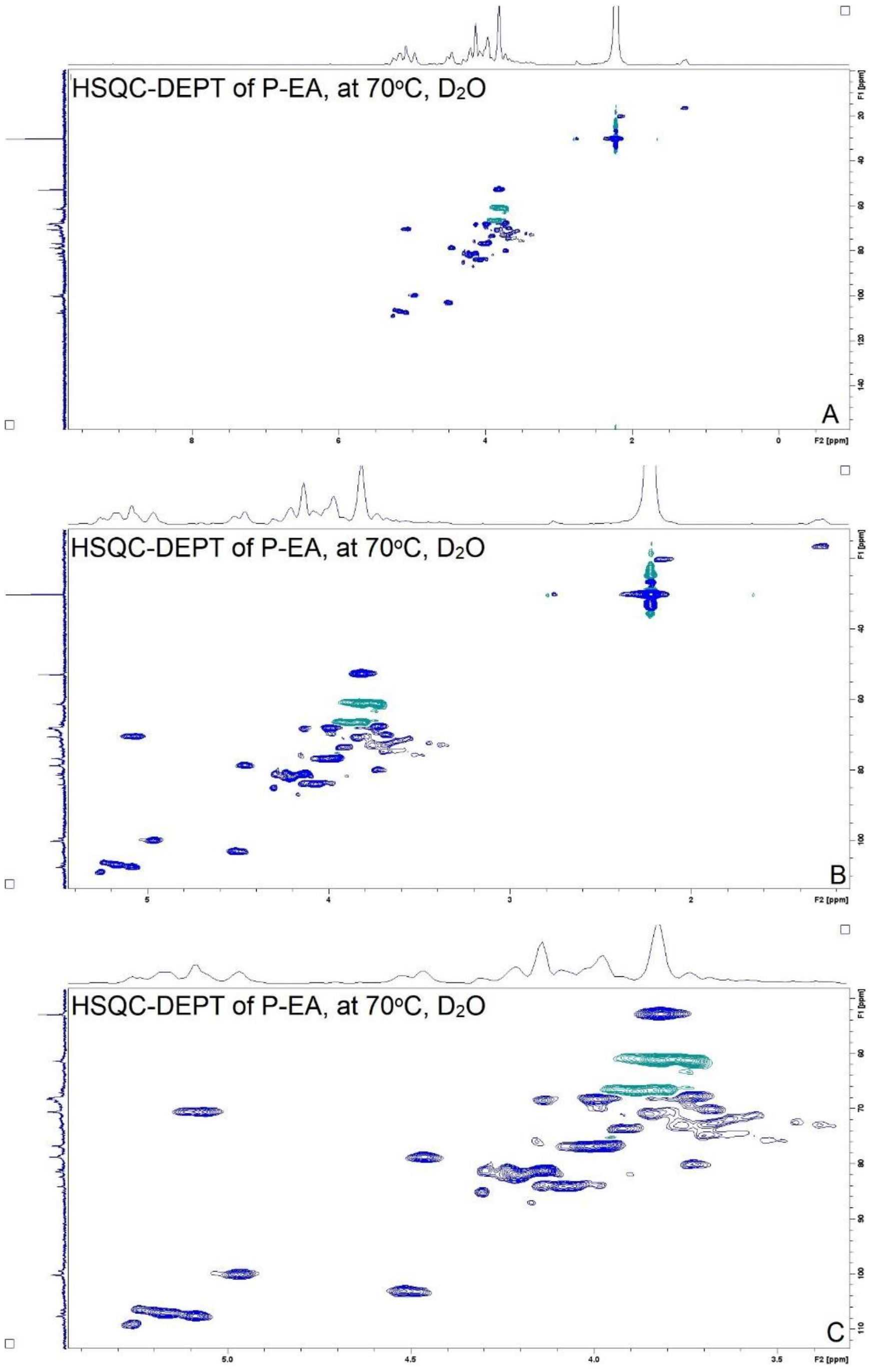

Figure S1: HSQC-DEPT correlation map of P-EA fraction in D_2O at 70°C, in different scales (A, B, and C). Inverted signals in DEPT experiment are marked in light green.
